# Specific and general adaptations following motor imagery practice focused on muscle strength in total knee arthroplasty rehabilitation: A randomized controlled trial

**DOI:** 10.1371/journal.pone.0221089

**Published:** 2019-08-14

**Authors:** Armin H. Paravlic, Rado Pisot, Uros Marusic

**Affiliations:** 1 Science and Research Centre, Institute for Kinesiology Research, Garibaldijeva 1, Slovenia; 2 Faculty of Sport, University of Ljubljana, Slovenia; 3 Department of Health Sciences, Alma Mater Europaea-ECM, Maribor, Slovenia; Universitat de Valencia, SPAIN

## Abstract

**Background:**

Motor imagery (MI) has been a widely used strategy in the past two decades to enhance physical capabilities among orthopaedic patients. However, its effectiveness is still questioned, since the demonstrated effects were likely task-dependent, with little evidence of transfer to tasks not specifically trained with MI.

**Objective:**

The aim of this study was to investigate whether an MI practice focused on maximal isometric knee extension strength, causes additional specific and general adaptations upon neuromuscular and functional variables when compared to conventional rehabilitation only, in patients submitted to total knee arthroplasty (TKA).

**Design:**

Parallel group randomized controlled clinical trial.

**Participants:**

Thirty-four patients (56% man) submitted to TKA.

**Program:**

Patients were randomly assigned to an MI practice group (MIp: 15 minutes per day/5 days per week in addition to routine physical therapy) or control group (CON) that performed routine physical therapy alone for four weeks.

**Outcome measures:**

The maximal isometric knee extension strength of the operated leg was defined as the primary outcome. Secondary outcomes were spatial and temporal gait parameters, 30-second chair sit-to-stand performance, a self-reported physical function assessed by the Lower Extremity Functional Scale (LEFS) questionnaire, and an MI ability score. All measurements were conducted before and one month after TKA.

**Results:**

Significant differences in treatment effects were observed for the MIp group compared to CON: the MIp showed less strength decrease (ES = 1.15, 95% CI: 0.32, 1.99, p = 0.022); faster self-selected speed under single (ES = 2.12, 95% CI: 1.16, 3.08, p = 0.001) and dual task (ES = 1.59, large, 95% CI: 0.67, 2.50, p = 0.002) conditions; brisk-pace gait speed during single (ES = 1.32, 95% CI: 0.47, 2.17, p = 0.020) and dual task conditions (ES = 1.31, large, 95% CI: 0.38, 2.23, p = 0.013); improved chair sit-to-stand (ES = 1.45, large, 95% CI: 0.58, 2.31, p = 0.004) performance; and a higher score on MI ability questionnaires for kinaesthetic imagery (KI) (ES = 0.55, 95% CI: -0.23, 1.34, p = 0.010) and internal visual imagery (EVI) (ES = 0.99, 95% CI: 0.18, 1.80, p = 0.039) scales, respectively. In addition, only MIp showed unaltered single and double support periods, as well as stride length and cadence during single task self-selected gait condition. Finally, analysis showed that the improved MI ability score achieved at the end of MI training was significantly correlated with the changes in the strength of the operated leg (kinaesthetic imagery: r = 0.741, p = 0.004; and internal visual imagery: r = 0.623, p = 0.023).

**Conclusions:**

MI training, when added in a corollary to routine physical therapy, led to improvements in both specific and general adaptations that were related to patients’ physical capabilities. While future studies must also evaluate the long-term effects, conducting MI training during acute and post-acute rehabilitation phases is advised, especially when the extent and range of physical exercise is limited or made impossible.

**Trial registration:**

ClinicalTrials.gov NCT03684148.

## Introduction

In the United States alone there are approximately 600,000 total knee arthroplasty (TKA) surgeries performed per year.[1] With an aging population and a greater prevalence of knee osteoarthritis (OA), by 2030 demands for TKAs are estimated to increase by 673%, posing a growing economic burden on modern societies.[2] Because of chronic OA, surgical trauma, and pain, TKA patients experience significant deterioration in both subjective and objective measurements of physical function, lasting even a few years after the surgery.[3,4]

Traditionally, post-TKA rehabilitation has consisted of a rather conventional approach to exercise that mechanically stresses the musculoskeletal system. Such exercise regimes have incorporated passive and/or active limb movements (aimed at improving the range of motion), restoration of gait, and strength and endurance exercises, and they have used both voluntary and electrically stimulated actions.[5,6] However, it is believed that motor impairments induced by surgery are largely driven by changes to the central (cortical and corticospinal) level of motor control, rather than the peripheral one (i.e. at the muscular level).[7–11]

There has been increasing interest over the past two decades in using cognitive-based strategies to improve motor skills.[10,12] One of the most popular types of non-physical exercises is motor imagery (MI), in which the patient mentally simulates a specific motor action without any actual corresponding motor output.[13] MI accordingly does not elicit pain or any other negative side effects in patients[14], nor does it require any special conditions, except a quiet space where the trainee/patient can relax and train. It is therefore an inexpensive and pragmatic application to use in rehabilitation when conventional exercise activities cannot be undertaken (e.g. due to injury, immobilization, or extensive pain). MI practice’s effectiveness relies on the functional equivalence theory, suggesting that similar neural networks are activated during imagining as during actual physical movement.[14,15] MI has been extensively investigated in a variety of symptomatic populations, with proven effectiveness in improving motor skills in conjunction with routine physical therapy (RPT).[16–19] For example, by implementing guided imagery in post-rehabilitation for TKA patients, Jacobson et al. (2016) found a positive transfer to gait velocity in comparison to the RPT group.[19] In combination with action observation of simple and complex gait patterns, MI training led to positive effects on task-specific outcomes following total hip arthroplasty (e.g., timed up-and-go test (TUG), four-step-square test, and fast-paced gait speed with the dual task).[17] Moreover, in the subsequent four weeks of MI practice combined with RPT, TKA patients demonstrated training-specific changes only (i.e., alleviated pain, improved range of motion, and increased knee extension strength), without effects beyond the imagined task such as reduction of knee swelling, or functional mobility assessed by a TUG test. In contrast to the aforementioned studies, a recent review with meta-analysis aimed to investigate the effects of MI on post-injury rehabilitation in athletes found only a small, insignificant effect on functional mobility recovery.[20] While the aforementioned studies substantially differed in design, and found inconsistent results regarding MI’s efficiency in the functional recovery of orthopaedic patients, it can be speculated that MI could serve as an upgrade of RPT for this specific population. Nevertheless, the demonstrated effects were likely task-dependent, with little evidence of their transfer to tasks not specifically trained with MI; this remains to be investigated.

A recent meta-analysis revealed that MI practice can be successfully implemented as a training tool in order to improve maximal voluntary strength in healthy adults.[21] Given that the knee extensors’ maximum strength was marked as a significant indicator of post-TKA patients’ physical capabilities,[22,23] we strove to investigate whether MI training that is focused on muscle strength only, in addition to RPT for rehabilitation of TKA patients, causes specific and general adaptations in neuromuscular and functional outcome measures at one month post-TKA. We therefore hypothesized that the MI practice group would experience less deterioration in: i) maximal isometric knee extension strength as a primary outcome; and ii) spatio-temporal gait parameters, lower body functional strength, and self-reported physical function as secondary outcomes.

## Materials and methods

This randomized controlled experimental trial was conducted according to the quidelines of the Consolidated Standards of Reporting Trials (CONSORT) Statement (S1 File).

### Trial design

We conducted this randomized controlled experimental trial at the Valdoltra Orthopaedic Hospital (Ankaran, Slovenia, EU) from August 2017 to June 2018. The clinical trial protocol was registered on ClinicalTrials.gov, Identifier: NCT03684148. The authors confirm that all ongoing and related trials for this trial have been registered. All procedures were carried out in accordance with the ethical standards of the 1964 Declaration of Helsinki and were approved by the Ethics Committee of Valdoltra Orthopaedic Hospital (approval No: 16/2016, issued on 7 December 2016). All patients provided informed, written consent. Following the initial recruitment of patients through the hospital’s general database, all eligible patients were equally randomized to one of two groups, namely, MI practice combined with the RPT group (MIp), or RPT only (CON). The participants were assessed at 1 day before the surgery (PRE) and at one month post-surgery (POST), with all measurements carried out in a separate, quiet room to avoid any external disturbances from the hospital environment.

#### Participants’ characteristics

**Fig 1:**
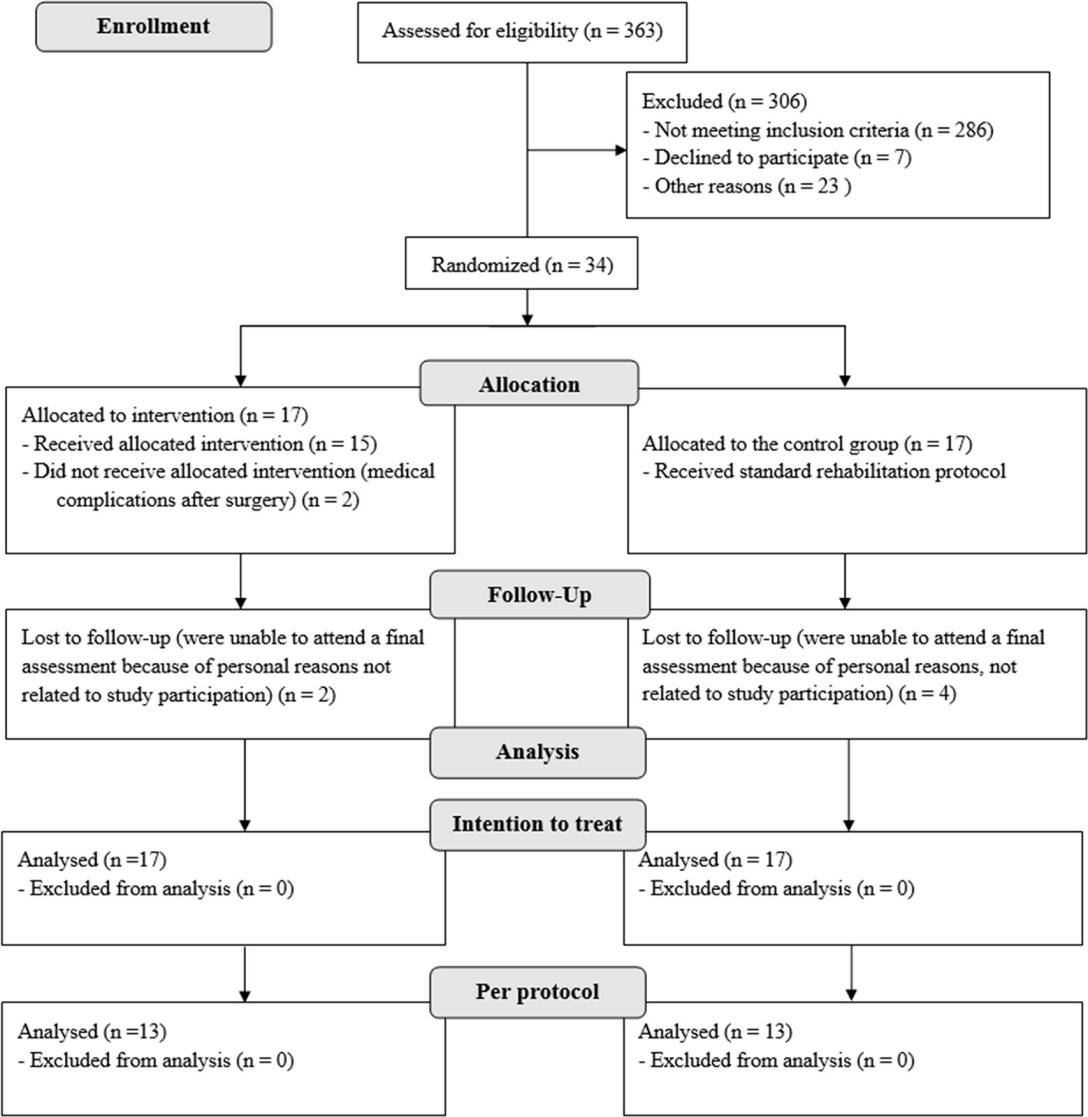
Flow chart of participant enrolment, randomized group allocation, and final analysis.

All patients underwent a tricompartmental, cemented TKA with a medial parapatellar surgical approach. The operations were conducted by three experienced surgeons. The enrolment, randomization, and final analysis procedures are shown in the CONSORT flow diagram (Fig 1). Patients were excluded if they were younger than 50 and/or older than 85 years of age, those scheduled for the revision of TKA; those who had bilateral TKAs; those with a body mass index (BMI) of 40 kg/m^2^ or higher; those with uncontrolled hypertension or diabetes mellitus; patients with a history of any neurological disorder including Cerebral Vascular Attack, Multiple Sclerosis, or Parkinson’s disease; patients with Rheumatoid Arthritis or active cancer; those with thrombosis; patients with contralateral knee OA (as defined by pain greater than 4/10 with activity, and presence of OA identified by radiography examination); those who had not been engaged in any pre-operative rehabilitation treatment; and those with any other unstable lower-extremity orthopaedic conditions after surgery, such as bleeding.

#### Sample size

Isometric knee extension strength of the involved leg was defined as the primary outcome variable for power analysis. The sample size was calculated prospectively using an available online spreadsheet (http://sportsci.org/resource/stats/index.html) in accordance with Hopkins recommendations.[24] We used unpublished data from our research group pilot study to calculate the raw mean difference in change (RDC) at RDC = 48.51 Nm. Further, the minimal clinically important change (MCIC = 21.89 Nm) was calculated from previously published data on the reliability of isometric muscle testing in TKA patients.[25] Given that it is not uncommon in clinical practice to lose patients during follow-up periods, this formula was adjusted in accordance to Sakpal recommendations,[26] which finally resulted in a sample size of 10 participants per group. In addition, a post-hoc sample size calculation was conducted based on the original data of the current study. Therefore, by using RDC of 33.40, new calculation resulted in 13 participants were needed per group.

#### Randomization and blinding

Patients were grouped based on a computer-generated list of random numbers (1 or 2) using Excel 2016 (Microsoft, Redmond, WA, USA). Group assignment took place after the inclusion criteria were met and prior to the preoperative testing session. Since financial resources did not permit the hiring of separate personnel for testing and MI practice treatments, the testers were not blinded to the patients’ group assignment, whereas all medical staff including nurses, physical therapists, and surgeons were unaware of the patients’ group allocation.

### Types of programs

#### Hospitalization period

Following the TKA, all patients spent an average of 6 days in the hospital (range 4–8 days). Regardless of their group assignment, all patients followed an identical inpatient rehabilitation program. The exercise program strove to improve patients’ general mobility via passive and active knee and hip flexion and extension exercises, muscle strengthening, walking with and without crutches, etc. At first, patients received 45 minutes of a continuous passive-motion session per day, conducted and monitored by certified medical personnel. Patients were further subjected to various functional everyday movement exercises, consisting of moving from laying to sitting with a transfer to a standing position and vice versa; training to walk with and without crutches, ascending/descending the stairs, weight-bearing exercises, etc. A detailed description of exercise program is available on ClinicalTrials.gov, Identifier: NCT03684148. A detailed description of the study protocol is available in both the original (Slovenian–S2 File) and English (S3 File) languages, respectively.

#### Home-based training

After being discharged from the hospital, both groups were supplied with a physical exercise program that they needed to perform at home consisting of the same exercise they had performed during inpatient care, with the exception of a continuous passive motion device, which they did not use in the home setting. To monitor their adherence to the prescribed exercise program, the patients were checked by phone.

#### Experimental group

In addition to RPT (inpatient rehabilitation), the patients from the MIp group were subjected MI training, beginning immediately after intensive care. They had been advised to imagine maximal voluntary isometric contractions (MViC). Accordingly, the patients performed two sets of 25 repetitions with 2 minutes of inter-set rest periods, for a duration of two weeks. To comply with the basic principles of strength training i.e., step-by-step overload and progression, 10 additional trials were added during weeks three and four, respectively. Each MViC repetition was sustained for 5 seconds, followed by a 5-second inter-repetition rest period. Additionally, after every fifth contraction, the participants had 20 seconds of rest. Following 5 days of MI practice, the participants were advised to take a break from MI for two consecutive days. On average, the patients performed three MI sessions during their hospitalization period. After hospital discharge, the participants from MIp group were supplied with an audio description (mp3 file) of MI session guidelines, which they continued at their homes. The MI script was structured as one training session (for example: two sets of 25 repetitions, for the first two weeks), consisting of information that should engage the patients in both kinaesthetic and visual MI. Patients were instructed to get comfortable in a regular chair and imagine the same set-up as during the MViC assessment. That is, they were to sit in a chair and imagine that the leg they were training was flexed 60° at the knee joint, while listening an audio tape with detailed practice instructions.

#### Control group

The patients from the control group followed the same post-surgery rehabilitation program as described above for five days a week (once per day) however, they were not subjected to MI practice. Each patient from the control group was contacted by phone on a weekly basis to ensure similar motivational levels in both groups. During each telephone call, the therapist asked the participants about their subjective health status, treatment adherence, and rehabilitation progress.

#### Treatment adherence

Regardless of group assignment, participants were called on a weekly basis to monitor their adherence to prescribed treatment for both the routine physical therapy and MI practice sessions.

### Measurement outcomes

Full list of outcome measures assessed in the current PhD project could be found here (S4 File).

### Primary outcomes

#### Maximal isometric knee extension strength

The maximal isometric knee extension strength of the operated leg was measured with a custom-build dynamometer (S2P Ltd., Bled, Slovenia), allowing for the proper positioning of participants based on the differences in their anthropometrics. In brief, the patients were seated in a dynamometer chair, with the hip fixed at 110° and the knee flexed at 60°. A steel cuff was strapped around the lower leg ~2 cm above the medial malleoli and connected to a strain-gauge load cell (Z6FC3–200 kg, HBM, Darmstadt, Germany), while the arms were crossed at the chest level. After the initial warm-up consisting of two submaximal contractions of approximately 50% and 75% of self-perceived maximal effort and one maximal voluntary isometric contraction (MViC) lasting up to three seconds, the patients were instructed to contract as fast and forcefully as possible and to maintain maximum force exertion until a plateau in force production was reached. All participants had up to three maximal trials, separated by 2 minutes of rest. During each trial, visual feedback and strong verbal encouragement were provided. The trial with the highest peak force was used as a representative MViC for further analysis. Isometric testing of knee extensor muscle strength showed high reliability in patients with TKA (with an interclass correlation coefficient of 0.965 and 0.950 for the involved and the uninvolved side, respectively).[25]

### Secondary outcomes

#### Gait measurements

Spatial and temporal gait parameters were measured with the 2D OptoGait system (Microgate, Bolzano, Italy) in the following four 1-minute conditions in a randomized order: 1) walking at their preferred, self-selected speed; 2) brisk walking to the best of their capacity; and 3–4) both speeds under a dual-task condition. In brief, for the self-selected speed condition, patients were instructed to walk as if they were going to walk to the nearest store and they were not in hurry. In contrast, for the brisk walking condition, patients were instructed to walk as if they were going to be late on the meeting or a bus. The dual-task conditions were composed of walking and at the same time subtracting by threes from a randomly chosen number between 400 and 500 (serial 3s). Before walking, all patients were subjected to a familiarization trial, after which they were administered 30-second baseline measurements during a subtraction task while standing quietly. The patients were instructed to subtract as many numbers as possible, with their focus prioritized on task correctness rather than speed of subtraction. The gait speed parameter was extracted for each condition using OptoGait software, version 1.11.1. For the cognitive task, the amount of subtracted numbers and errors was monitored for each condition and, finally, the number of correct answers was further analysed.

#### Chair sit-to-stand

The chair sit-to-stand movement test is used to assess lower body strength, which is necessary for several everyday tasks such as climbing stairs, walking, and getting out of a chair, etc. Additionally, it is a valid test to measure patients’ functional recovery following TKA surgery.[27] Each subject completed two practice repetitions and one 30-second test trial. The final score was the total number of times they could stand up correctly within 30 seconds.[28]

#### Self-reported physical function

The Lower Extremity Functional Scale (LEFS) questionnaire, translated into Slovenian, was used to evaluate the self-reported functional impairment of patients with lower-extremity musculoskeletal dysfunction.[29] Briefly, the questionnaire includes 20 items that are related to the person’s difficulty in performing everyday activities such as usual work or housework, squatting, lifting objects, rolling over in bed, etc. Each answer is scaled from 0 (extreme difficulty or unable to perform activity) to 4 (no difficulty). The higher the result on the test, the greater the subject’s physical capabilities.

#### Motor imagery ability

For the purpose of this study, we used a valid version of the motor imagery questionnaire 3 (MIQ-3) that was translated into Slovenian.[30] The MIQ-3 is a questionnaire consisting of a total of 12 items aimed at assessing an individual’s ability to imagine four movements such as leg raise, jump, arm abduction and adduction, and standing hip flexion using visual i.e., internal visual imagery (IVI) or external visual imagery (EVI), along with kinaesthetic imagery (KI).[30]

### Data analysis

The data were analysed using IBM SPSS Statistics 24.0 software for Windows (SPSS Inc., Chicago, Il, USA). A one-way analysis of variance (ANOVA) was used to examine whether participants who completed the study protocol differed from those who did not (i.e., drop outs N = 8).

To minimise the results of interpretation bias due to patients lost to follow up, both intention-to-treat and per-protocol analysis were utilized as recommended.[31,32] Therefore, a mixed effect models were used to examine the primary and secondary hypotheses using intention-to-treat approach, allowing us to incorporate all patients who were originally randomly assigned to their groups (including data from patients with missing values). The maximum likelihood estimation method for missing values was used in the mixed effects models. Missing data (N = O from baseline; N = 8 from final measurements) was not imputed. In all models, the fixed factor was group assignment (MIp vs. CON), with two-time points (PRE vs. POST). In addition, a sensitivity analysis was used to investigate the true effect of treatment by excluding participants who were lost during follow up (N = 8); consequently 26 patients remained for per-protocol analysis. No differences in patterns were observed, thus Cohen’s *d* effect size (ES) were also calculated on a per-protocol approach basis to determine the magnitude of the group’s differences in primary and secondary outcomes.[33] The magnitude of ES was interpreted as follows: *trivial* = <0.20; *small* = 0.2–0.59; *moderate* = 0.60–1.19; *large* = 1.20–1.99; and *very large* = >2.0 based on Hopkins recommendations [34]. Further, magnitude-based inferences of observed ES were determined and interpreted qualitatively as: *almost certainly not* = <0.5%; *very unlikely* = 0.5%-5%; *unlikely* = 5%-25%; *possible* = 25%-75%; *likely* = 75%-95%; *very likely* = 95%-99.5%; and *almost certain* = >99.5%.[34] In the case of significant interactions, post hoc comparisons with Bonferroni corrections were applied. Additionally, the percentual relationship between the change ([postoperative value-preoperative value]/preoperative value) in maximal isometric knee extension strength and patients’ motor imagery ability was examined using Pearson’s Correlation Coefficient. Statistical significance was set at the level of p < 0.05. The ES should be interpreted with caution, namely the direction of ES. For example, the spatiotemporal gait parameters during self-selected pace gait (i.e., the single support period and double support period) have a negative direction. However, these changes are actually beneficial because of less time spent in these phases of walking results in improvement of gait speed.

## Results

### Study population

In total, 34 patients (19 males and 15 females) were randomized (Table 1), whereas 26 patients completed both PRE and POST measurements (details depicted in Fig 1). For per-protocol analysis all patients (MIp group N = 13, CON group N = 13) completed all measurements, except for the dual-task gait condition. Namely, two patients (one per group) did not complete the self-selected pace gait with dual task, three patients from the CON group were physically unable to complete the brisk-pace gait under the dual-task condition and, finally, one patient from the MIp group did not want to participate in dual task conditions due to unpleasant feelings as a result thereof.

**Table 1:** Participants' characteristics baseline.

	Total Sample	MIp group (N = 17)	Control group (N = 17)
**Demographic Characteristics**			
Age (years)	61.1 ± 5.3	62.2 ± 4.9	60.0 ± 5.7
Sex (men/women)	19/15	7/6	7/6
BMI (kg/m^2^)	30.1 ± 3.2	29.7 ± 4.2	30.4 ± 1.80
Ethnicity	White/South Slavic (100%)	White/South Slavic (100%)	White/South Slavic (100%)
Total knee arthroplasty, (right side, n)	19/34	9/17	10/17
Days of hospital stay after TKA	6.5 ± 1.4 (range, 4–8)	6.4 ± 1.5 (range, 4–8)	6.7 ± 1.3 (range, 5–8)
**Physical function**			
Maximal isometric extension strength of the operated knee (Nm)	125.8 ± 47.9	117.3 ± 48.6	134.3 ± 47.1
30s Chair sit to stand (n)	10.4 ± 3.0	9.9 ± 2.8	10.8 ± 3.3
Self-selected gait speed (m/s)	1.1 ± 0.2	1.1 ± 0.2	1.2 ± 0.1
Self-selected gait speed DT (m/s)	1.0 ± 0.2	1.0 ± 0.2	1.1 ± 0.2
Brisk-paced gait speed (m/s)	1.4 ± 0.2	1.5 ± 0.2	1.4 ± 0.2
Brisk paced gait speed DT (m/s)	1.3 ± 0.2	1.2 ± 0.2	1.4 ± 0.2
**Spatiotemporal gait parameters during self-selected pace gait**			
Single support period (s)	0.4 ± 0.0	0.4 ± 0.0	0.4 ± 0.0
Double support period (s)	0.3 ± 0.1	0.3 ± 0.1	0.3 ± 0.0
Stride length (cm)	128.2 ± 13.8	126.7 ± 13.8	129.7 ± 14.2
Cadence (steps/min)	105.1 ± 9.7	101.7 ± 11.2	108.6 ± 6.5
**Self-reported physical function and motor imagery ability**			
LEFS (points)	32.2 ± 7.6	33.1 ± 7.0	31.5 ± 8.4
Kineasthetic imagery ability	5.5 ± 1.2	5.6 ± 0.8	5.4 ± 1.5
Internal visual imagery ability	5.7 ± 1.2	5.6 ± 1.3	5.9 ± 1.0
External visual imagery ability	5.9 ± 0.9	5.9 ± 1.1	5.9 ± 0.8
**Cognitive performance**			
Baseline (number of correct answers)	24.4 ± 8.1	24.8 ± 9.4	24.0 ± 6.9
Serial 3s during self-selected gait speed (number of correct answers)	41.5 ± 15.8	42.4 ± 14.4	40.7 ± 17.6
Serial 3s during brisk-paced gait (number of correct answers)	46.2 ± 17.6	46.8 ± 16.3	45.5 ± 19.3

*Note*: Data are presented as mean ± SD

*BMI–*body mass index; *MIp–*motor imagery practice group; *TKA–*total knee arthroplasty; *LEFS* Lower extremity functional score questionnaire

#### Adherence to training

During the study period, all patients included in MIp group completed 20 MI sessions, of 9 minutes in duration on average. The adherence to the prescribed MI post-rehabilitation was as high as 98%. The adherence to RPT was high, that is 98% and 96% for MIp and CON group, respectively.

#### Tailoring and modifying training

There were no serious negative side effects reported during the course of the study that were related to the prescribed training program. Neither of the prescribed programmes, i.e. MI and RPT, were modified during the course of the study.

### Primary outcome

#### Maximal isometric knee extension strength

A significant time x group interaction effect (F_1,34_ = 4.721, p = 0.037) was found for the maximal isometric knee extension strength (Table 2). Post-hoc tests revealed that, compared to the CON group, MIp experienced significantly lower deterioration in strength one month after the surgery (ES = 1.15, moderate, 95% CI: 0.32, 1.99, p = 0.022) (Table 3).

**Table 2:** Mixed effects models for physical performance and self-reported measures (intention to treat analysis).

	MIp group			Control group			LMEM time effect		time*group effect	t value (*p* value)
	PRE	POST	Δ (%)	PRE	POST	Δ (%)	Parameter Estimate (SE)	t value (*p* value)	Parameter Estimate (SE)	
**Physical function**										
Maximal isometric extension strength of the operated knee (Nm)	117.3 ± 48.6	68.1 ± 32.3	-41.9	134.3 ± 47.1	43.3 ± 25.1	-67.8	101.2 (11.7)	8.637 (**<0.001**)	-36.0 (16.6)	-2.173 (**0.037**)
30s Chair sit to stand (n)	9.9 ± 2.8	9.2 ± 2.6	-7.4	10.8 ± 3.3	5.8 ± 2.7	-46.7	5.0 (0.8)	6.608 (**<0.001**)	-4.1 (1.1)	-3.833 (**<0.001**)
Self-selected gait speed (m/s)	1.1 ± 0.2	1.1 ± 0.1	3.5	1.2 ± 0.1	0.9 ± 0.2	-25.0	0.3 (0.1)	5.382 (**<0.001**)	-0.3 (0.1)	-4.301 (**<0.001**)
Self-selected gait speed DT (m/s)	1.0 ± 0.2	1.0 ± 0.2	2.8	1.1 ± 0.2	0.9 ± 0.2	-22.6	0.3 (0.1)	4.821 (**<0.001**)	-0.3 (0.1)	-3.709 (**0.001**)
Brisk-paced gait speed (m/s)	1.5 ± 0.2	1.4 ± 0.2	-3.3	1.4 ± 0.2	1.0 ± 0.3	-28.2	0.4 (0.1)	5.052 (**<0.001**)	-0.4 (0.1)	-3.210 (**0.003**)
Brisk-paced gait speed DT (m/s)	1.2 ± 0.2	1.2 ± 0.2	-3.0	1.4 ± 0.2	1.0 ± 0.3	-27.6	0.4 (0.1)	5.192 (**<0.001**)	-0.3 (0.1)	-3.328 (**0.003**)
**Spatiotemporal gait parameters during self-selected pace gait**										
Single support period (s)	0.4 ± 0.0	0.4 ± 0.1	-3.5	0.4 ± 0.0	0.4 ± 0.0	7.5	-0.03 (0.1)	-3.020 (**0.005**)	0.05 (0.02)	3.264 (**0.003**)
Double support period (s)	0.3 ± 0.1	0.3 ± 0.1	-7.7	0.3 ± 0.0	0.4 ± 0.1	35.4	-0.11 (0.02)	-4.614 (**<0.001**)	0.13 (0.03)	4.084 (**<0.001**)
Stride length (cm)	126.7 ± 13.8	126.4 ± 12.0	-0.2	129.7 ± 14.2	111.0 ± 17.2	-14.5	17.5 (2.9)	6.010 (**<0.001**)	-16.3 (4.1)	-3.974 (**<0.001**)
Cadence (steps/min)	101.7 ± 11.2	105.9 ± 8.5	4.1	108.6 ± 6.5	94.8 ± 7.9	-12.7	13.7 (3.2)	4.275 (**<0.001**)	-17.8 (4.5)	-3.928 (**<0.001**)
**Self-reported physical function and motor imagery ability**										
LEFS (points)	33.1 ± 7.0	34.1 ± 6.8	3.1	31.5 ± 8.4	26.2 ± 5.6	-16.6	5.1 (2.2)	2.298 (**0.030**)	-6.4 (3.1)	-2.027 (0.053)
Kineasthetic imagery ability	5.6 ± 0.8	5.6 ± 0.8	0.0	5.4 ± 1.5	5.1 ± 1.0	-5.9	0.5 (0.1)	3.534 (**0.002**)	-0.5 (0.2)	-2.675 (**0.013**)
Internal visual imagery ability	5.6 ± 1.3	5.7 ± 0.8	2.8	5.9 ± 1.0	5.4 ± 0.6	-8.7	0.5 (0.2)	2.309 (**0.028**)	-0.7 (0.3)	-2.207 (**0.035**)
External visual imagery ability	5.9 ± 1.1	5.8 ± 0.8	-0.6	5.9 ± 0.8	5.8 ± 0.5	-2.0	0.1 (0.2)	0.558 (0.581)	-0.1 (0.3)	-0.488 (0.629)
**Cognitive performance**										
Baseline (number of correct answers)	24.8 ± 9.4	29.5±8.9	19.1	24.0 ± 6.9	27.5±10.9	14.6	-4.6 (1.8)	-2.554 (**0.017**)	0.5 (2.5)	0.212 (0.834)
Serial 3s during self-selected gait speed (number of correct answers)	42.4 ± 14.4	50.3±17.7	18.8	40.7 ± 17.6	42.5±18.4	4.4	-5.2 (2.7)	-1.923 (0.066)	-1.2 (3.8)	-0.320 (0.752)
Serial 3s during brisk-paced gait (number of correct answers)	46.8 ± 16.3	53.8±19.1	15.0	45.5 ± 19.3	38.4±13.0	-15.7	-0.7 (3.3)	-0.226 (0.823)	-3.7 (4.4)	-0.826 (0.418)

***Note*:** Data are presented as mean ± SD; ***Bolded*** values represents a significant effect

*MIp* motor imagery practice group; *LEFS* Lower extremity functional score questionnaire; *Δ* (%)–percent changes between initial and final measurement; SE–standard error; *LMEM–*Linear mixed effects models

**Table 3:** Magnitude-based inferences and absolute changes between initial and final measurements in: Motor imagery group, control group, and between groups (per protocol analysis).

	Motor Imagery group		Control group		Between group comparison		
	Raw diff (95% CI)	ES (95% CI)	Raw diff (95% CI)	ES (95% CI)	Raw diff (95% CI)	ES (95% CI)	MBI
**Physical function**							
Maximal isometric extension strength of the operated knee (Nm)	-49.70 (-78.42, -20.98)	-1.33 (-2.39, -0.28)	-83.10 (-113.56, -52.64)	-2.10 (-3.47, -0.72)	33.40 (-8.46, 75.26)	1.15 (0.32, 1.99)	likely beneficial
30s Chair sit to stand (n)	1.10 (-3.22, 1.02)	-0.40 (-1.20, 0.40)	4.92 (-7.24, -2.60)	-1.63 (-2.80, -0.46)	3.82 (0.68, 6.96)	1.45 (0.58, 2.31)	very likely beneficial
Self-selected gait speed (m/s)	0.04 (-0.08, 0.16)	0.26 (-0.52, 1.04)	-0.29 (-0.41, -0.17)	-1.92 (-3.21, -0.62)	0.33 (0.16, 0.50)	2.12 (1.16, 3.08)	most likely beneficial
Self-selected gait speed DT (m/s)	0.01 (-0.13, 0.15)	0.06 (-0.74, 0.86)	-0.26 (-0.40, -0.12)	-1.53 (-2.70, -0.35)	0.27 (0.07, 0.47)	1.59 (0.67, 2.50)	very likely beneficial
Brisk-pace gait speed (m/s)	-0.08 (-0.23, 0.07)	-0.40 (-1.20, 0.40)	-0.38 (-0.57, -0.19)	-1.55 (-2.69, -0.41)	0.30 (0.06, 0.54)	1.32 (0.47, 2.17)	very likely beneficial
Brisk-pace gait speed DT (m/s)	-0.07 (-0.21, 0.07)	-0.39 (-1.22, 0.44)	-0.35 (-0.54, -0.16)	-1.61 (-2.93, -0.28)	0.28 (0.05, 0.51)	1.31 (0.38, 2.23)	very likely beneficial
**Spatiotemporal gait parameters during self-selected pace gait**							
Single support period (s)	-0.02 (-0.05, 0.01)	-0.44 (-1.25, 0.36)	0.03 (0.00, 0.06)	0.85 (-0.05, 1.75)	-0.05 (-0.09, -0.01)	-1.25 (-2.09, -0.41)	very likely beneficial
Double support period (s)	-0.03 (-0.36, 0.30)	-0.07 (-0.84, 0.70)	0.10 -(0.39, 0.59)	0.16 (-0.62, 0.93)	-0.13 (-0.72, 0.46)	-0.18 (-0.95, 0.59)	possibly trivial
Stride length (cm)	-1.40 (-10.86, 8.06)	-0.11 (-0.89, 0.66)	-17.10 (-29.12, -5.08)	-1.09 (-2.07, -0.12)	15.70 (0.40, 31.00)	1.06 (0.24, 1.88)	likely beneficial
Cadence	5.30 (-2.13, 12.73)	0.55 (-0.28, 1.37)	-14.30 (-20.00, -8.60)	-1.93 (-3.23, -0.63)	19.60 (10.24, 28.96)	2.39 (1.38, 3.39)	most likely beneficial
**Self-reported physical function and motor imagery ability**							
LEFS (points)	2.46 (-2.83, 7.75)	0.36 (-0.44, 1.15)	-4.46 (-8.85, -0.07)	-0.78 (-1.66, 0.10)	6.92 (0.05, 13.79)	1.11 (0.29, 1.94)	likely beneficial
Kineasthetic imagery ability	0.10 (-0.55, 0.75)	0.12 (-0.65, 0.89)	-0.40 (-1.21, 0.41)	-0.38 (-1.18, 0.42)	0.50 (-0.54, 1.54)	0.55 (-0.23, 1.34)	possibly beneficial
Internal visual imagery ability	0.20 (-0.63, 1.03)	0.19 (-0.59, 0.96)	-0.50 (-1.04, 0.04)	-0.71 (-1.57, 0.15)	0.70 (-0.29, 1.69)	0.99 (0.18, 1.80)	likely beneficial
External visual imagery ability	0.10 (-0.64, 0.84)	0.10 (-0.67, 0.87)	-0.10 (-0.57, 0.37)	-0.16 (-0.94, 0.61)	0.20 (-0.67, 1.07)	0.30 (-0.47, 1.07)	possibly trivial
**Cognitive performance**							
Baseline (number of correct answers)*	4.90 (-2.42, 12.22)	0.51 (-0.30, 1.33)	5.20 (-1.79, 12.19)	0.60 (-0.27, 1.46)	-0.30 (-10.45, 9.85)	-0.03 (-0.81, 0.75)	likely trivial
Serial 3s during self-selected gait speed (number of correct answers)*	8.00 (-5.08, 21.08)	0.47 (-0.34, 1.28)	6.30 (-7.68, 20.28)	0.36 (-0.47, 1.19)	1.70 (-17.43, 20.83)	0.09 (-0.69, 0.88)	likely trivial
Serial 3s during brisk-paced gait (number of correct answers)*	5.80 (-7.74, 19.34)	0.33 (-0.46, 1.12)	-1.60 (-15.06, 11.86)	-0.10 (-0.98, 0.77)	7.40 (-12.06, 26.86)	0.44 (-0.39, 1.28)	possibly trivial

*95% CI* 95% confidence interval; *LEFS* Lower extremity functional score questionnaire; *DT* dual task; *ES* effect size; *MBI* magnitude-based inferences interpretation

*Number of patients per outcome measure are mentioned in a first paragraph of results section.

### Secondary outcomes

#### A 30 second chair stand test

Both the MIp and CON groups performed significantly worse in the chair sit-to-stand test one month after surgery compared to PRE (F_1,30.30_ = 30.396, p < 0.001). However, there was a significant time x group interaction effect (F_1,30.30_ = 14.688, p = 0.001). Post-hoc analysis showed that the MIp group experienced a significantly lower decrease in the chair sit-to-stand performance compared to the CON group (ES = 1.45, large, 95% CI: 0.58, 2.31, p = 0.004) (Table 3).

#### Self-reported physical function

There was no significant time x group interaction effect (F_1,25.65_ = 4.108, p = 0.053), thus a post-hoc analysis was not performed.

### Motor imagery ability

Significant time x group interaction was observed for two dimensions of motor imagery one month after the surgery (KI: F_1,24.85_ = 7.7153, p = 0.013; IVI: F_1,29.42_ = 4.869, p = 0.035), except for EVI (F_1,31.67_ = 0.239, p = 0.629). Post-hoc comparisons showed that MI group experienced a significant improvement in KI (ES = 0.55, small, 95% CI: -0.23, 1.34, p = 0.010) and IVI (ES = 0.99, moderate, 95% CI: 0.18, 1.80, p = 0.039) without significant alterations in EVI (ES = 0.30, small, 95% CI: -0.47, 1.07, p = 0.504) (Table 3). Additional correlation analysis showed that changes in KI (r = 0.741; p = 0.004) and IVI (r = 0.623; p = 0.023) significantly correlated with changes in the maximal isometric knee extension strength of the operated leg from pre to post (Table 4).

**Table 4:** Correlations between maximal isometric knee extension strength and motor imagery ability during PRE and for calculating the % of change.

Independent variables	Knee extensors strength (PC)
	r (p value)
Kineasthetic imagery ability (PRE)	-.021 (.946)
Internal visual imagery ability (PRE)	-.445 (.128)
External visual imagery ability (PRE)	-.411 (.163)
Kineasthetic imagery ability (%change)	**.741 (.004)**
Internal visual imagery ability (%change)	**.623 (.023)**
External visual imagery ability (%change)	.327 (.276)

*Note*: ***Bolded*** values represents a significant effect

*PRE*–measurement at baseline, PC–percent of change from pre to post; *r*–Person correlation coefficient.

### Gait speed parameter under single- and dual-task conditions

#### Gait speed during self-selected gate

**Fig 2:**
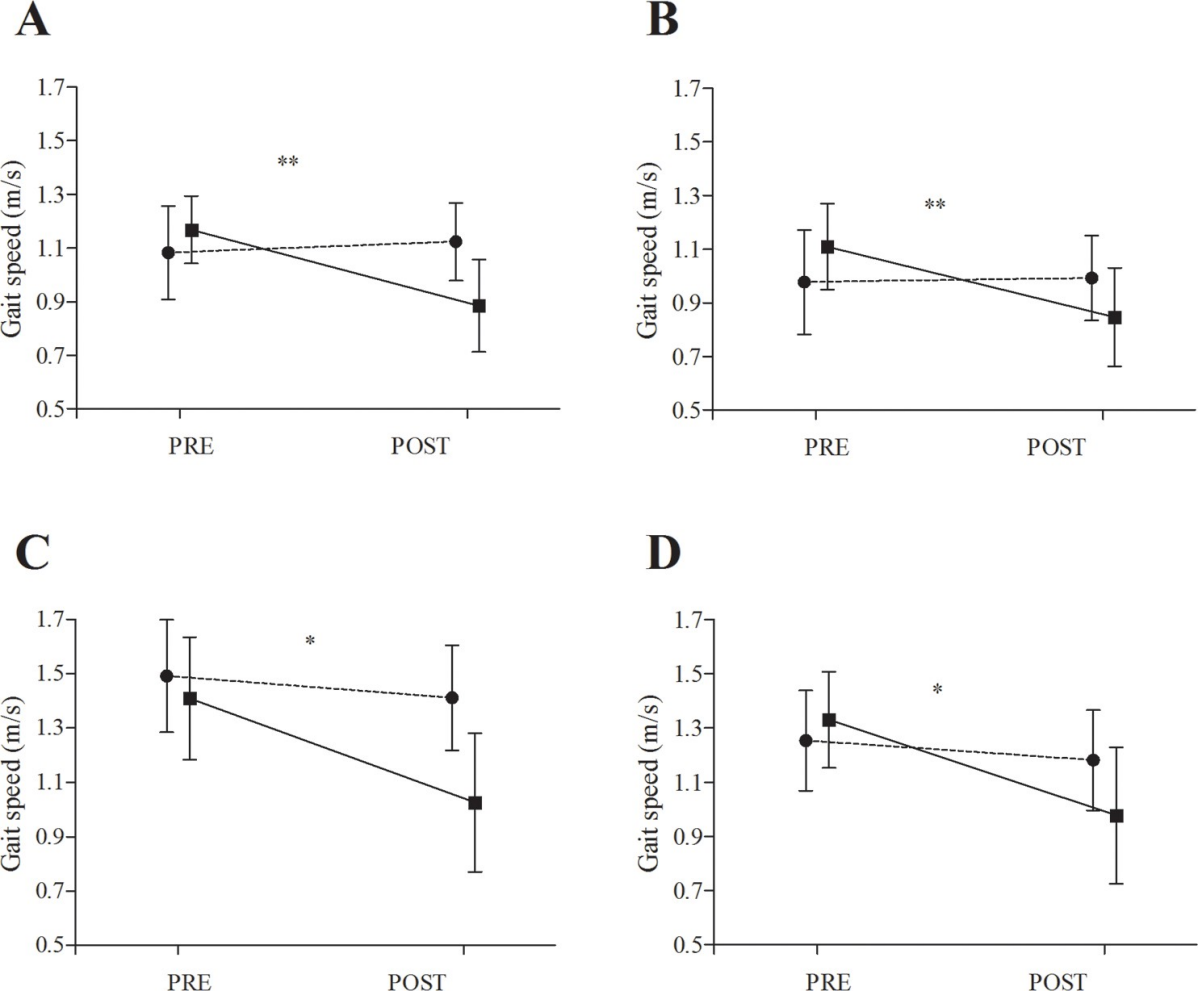
**Illustration showing gait speed results during walking in: a) self-selected pace under single-task condition; b) self-selected pace under dual-task condition; c) brisk-pace under single-task condition and d) brisk-pace under dual-task condition.** Data are presented as means and standard deviations. * indicates a significant change of p< 0.05; ** indicates a significant change of p< 0.01 from PRE to POST, respectively.

A significant time x group interaction effect (F_1,27.92_ = 18.501, p < 0.001) for the self-selected gait speed parameter in single-task condition. Post-hoc tests revealed that the MIp group experienced a significantly lower deterioration in gait speed than the CON group (ES = 2.12, very large, 95% CI: 1.16, 3.08, p = 0.001) (Fig 2A).

Furthermore, there was also a significant time x group interaction effect (F_1,27.66_ = 13.756, p = 0.001) for the self-selected gait speed parameter under the dual-task condition where MIp group experienced significantly lower deterioration in gait speed as compared to the CON group (ES = 1.59, large, 95% CI: 0.67, 2.50, p = 0.002) (Fig 2B).

#### Gait speed during brisk-pace

A significant time x group interaction effect (F_1,29.71_ = 10.714, p = 0.003) was observed for the gait speed during the brisk-pace under the single-task condition. Post-hoc tests revealed that the MIp group experienced a significantly lower decrease in gait speed as compared to the CON group (ES = 1.32, large, 95% CI: 0.47, 2.17, p = 0.020) (Fig 2C).

Furthermore, there was also a significant time x group interaction effect (F_1,25.22_ = 11.074, p = 0.003) also for the gait speed during brisk-pace in dual-task condition, where MIp group experienced a significantly lower decrease in gait speed as compared to CON group (ES = 1.31, large, 95% CI: 0.38, 2.23, p = 0.013) (Fig 2D).

#### Single support period, double support period, stride length, and cadence during self-selected gait

A significant time*group interaction was observed for both the single (F_1,30.20_ = 10.651, p = 0.003) and double (F_1,30.65_ = 16.681, p < 0.001) support phases. Post-hoc tests revealed that the MIp group spent significantly less time in both single (ES = -1.25, large, 95% CI: -2.09, -0.41, p = 0.003) and double support (ES = -0.18, trivial, 95% CI: -0.95, 0.59, p = 0.001) at POST, while no changes were observed in the exercising group (both p ≥ 0.183). Further, a significant time x group interaction effect was observed for stride length (F_1,26.44_ = 15.789, p < 0.001). Post-hoc analyses revealed that there were no significant alterations in stride length in MIp group (ES = -0.11, trivial, 95% CI: -0.89, 0.66, p = 0.673), while the CON group experienced a significant reduction at POST (ES = -1.09, moderate, 95% CI: -2.07, -0.12, p < 0.001). A significant time x group interaction effect was also observed for the gait cadence (F_1,30.94_ = 15.431, p < 0.001). Post-hoc analysis showed significant deterioration of gait cadence for the CON group only (ES = -1.93, large, 95% CI: -3.23, -0.63, p < 0.001), without a significant decrease in MIp (ES = 0.55, small, 95% CI: -0.28, 1.37p = 230) (Table 3).

### Cognitive performance during baseline (standing), self-selected, and brisk-pace gait dual-task trials

Performance during serial subtraction by threes under standing and gait conditions varied substantially and are summarized in Table 3. There were no significant time x group interaction effect was observed (F_1,24.06_ = 0.045, p = 0.834). On the contrary, there was a significant main effect (F_1,24.06_ = 11.564, p = 0.002) where both the MIp (ES = 0.51, small, 95% CI: -0.30, 1.33, p = 0.029) and CON (ES = 0.60, moderate, 95% CI: -0.27, 1.46, p = 0.050) groups gave more correct answers POST than PRE. Similarly, during the self-selected dual-task gait condition no significant time x group interaction effect was observed (F_1,24.15_ = 0.102, p = 0.752). A significant main effect (F_1,24.15_ = 9.243, p = 0.006) was observed where only the MIp group showed significant improvements during POST as compared to PRE (ES = 0.47, small, 95% CI: -0.34, 1.28, p = 0.023). Improvements in the CON group were not significant (ES = 0.36, small, 95% CI: -0.47, 1.19, p = 0.116). Finally, the study also failed to find any significant time x group interaction (F_1,22.05_ = 0.682, p = 0.418), nor was there a main effect (F_1,22.05_ = 1.344, p = 0.259) observed during serial subtraction-by-threes tasks under the brisk-pace dual-task gait condition (Table 2).

## Discussion

The findings from the present study suggest that MI training, in addition to RPT, was effective in enhancing patients’ rehabilitative outcomes following TKA. Results showed that MI based solely on strength tasks reduced the deterioration of maximal isometric knee extension strength and, more importantly, the proposed training regime has positive transfer effects on several other functional outcomes, such as gait speed under single and dual task conditions, and the repetitive strength of lower limbs one month after the surgery. In addition, correlation analysis showed that changes in MI ability score, experienced at the end of the MI training, significantly correlated with changes on the strength of the operated leg. Moreover, the observed changes were specific to the imagery’s modality, given that only kinaesthetic and internal visual imagery scores positively correlated with changes in strength after MI training. Finally, considering chosen variables evaluating the cognitive performance, the current study failed to prove the efficiency of MI training when compared to the conventional rehabilitation practice.

An extensive amount of literature postulates a loss in quadriceps strength following muscle disuse due to injury or/and surgery,[35–38], where the major moderating factors behind these phenomena were prescribed to adaptations on the neural components of motor control, observed on both the corticospinal [9,10] and peripheral levels.[36,39] Given that TKA patients were unable to undertake conventional strength training immediately after their surgery, MI training was administered in addition to RPT. To the best of our knowledge, only one study had previously examined the applicability of MI training in TKA patient rehabilitation.[40] In contrast to the present study, previously-administered MI training consisted of a multi-objective approach, aimed at addressing different rehabilitation outcomes, e.g. knee pain, strength, and range of motion, while imagery content and modalities varied, depending on particular session recovery goals.[40] Authors have found task-specific effects only, i.e. improved knee extension strength and range of motion, and reduced pain, however, edema and functional mobility assessed by timed up-and-go test were not significantly altered after MI practice.[40] In line with previous findings, our study showed improvements in knee extension strength, suggesting that MI training could be used effectively to increase strength in patients with peripheral locomotor system damage.[40,41] When one mentally simulates forceful muscle contractions, brain activity increases, leading to greater descending command to agonistic muscle and, consequently, motor output increases.[42–44] Accordingly, the effectiveness of MI training in improving physical function might be explained merely through neural adaptations,[43,45] knowing that there is no structural muscle change.[46] These alterations in the central neural network were shown to be a persons’ imagery ability, dependent along with the type of perspective or imagery modality used.[47,48] Previously it was reported that similar brain activation patterns were seen when performing an action and imagining it;[15,42,49] however, there is evidence that kinaesthetic and visual imagery differ in brain activation, whereas visual imagery showed the involvement of primary visual areas such as the occipital regions and the superior parietal lobules.[49] In contrast, kinaesthetic imagery showed stronger activation of many motor- related frontal areas, bilateral basal ganglia, and the cerebellar hemispheres.[49] Moreover, the studies by Stinear et al. (2006) showed a modulation of corticospinal excitability following only kinaesthetic imagery,[50] which consequently translates to a greater improvement in motor performance.[51,52] The current study found a high level of patients’ MI ability for all three perspectives at baseline. As expected, the treatment has a positive influence on the MI ability of the group that trained only, which significantly correlates with improvements in the MI group’s strength from-pre to post. Therefore, the present findings suggest that imagery ability, particularly kinaesthetic and internal visual perspectives, could be improved following MI treatment.[53] These observations were probably the reflections of imagery script that was constructed to encourage patients to feel kinaesthetic sensations during a practice.

It is well known that patients with knee OA have problems with their gait. Both before and after TKA they tend to walk more slowly than healthy control groups.[54] A recent meta-analysis actually failed to show the positive alterations of different treatments on gait speed for a period of up to five months after TKA[55]¸ which was due to a large amount of heterogeneity between included studies.[55] Moreover, the study aimed to compare three different programs targeting functional performance following TKA that showed non-significant differences in self-selected gait speed improvements afterwards (-0.34m/s), aquatic (-0.22m/s), and water exercise (-0.27m/s) at two weeks postoperatively, respectively.[56] In the present study, the MI training group proved to be superior in reducing the detrimental effects of TKA on gait speed when compared to RPT at one month postoperatively (MIp: +0.04m/s vs., CON: -0.29m/s; self-selected gait speed). The grater preservation of pre-operative quadriceps strength, i.e. less asymmetry between limbs, could be directly related to gait and chair sit-to-stand performance following surgery.[57] Further, a very likely beneficial effect was observed for dual-task conditions in the MIp group compared to CON. In everyday activities people commonly confront situations where postural adjustments and/or gait are overlapped with additional cognitive motor activity, such as talking, texting, avoiding obstacles, or performing other cognitively-challenging tasks that require allocating attention between concurrent demands. This concurrent activity, called dual task, has garnered growing interest, focusing on aging and neurodegenerative diseases.[58,59] Studies showed that dual task performance affects gait in both healthy and symptomatic older adults.[58–60] Toulotte et al.[60] showed that healthy elderly citizens who fall, as compared to those who do not fall, experienced a reduction in gait speed, cadence, stride, and step length, whereas step time and single support time were longer under dual task conditions than under single task. Similarly, the results from spatiotemporal gait characteristics revealed that the superiority of MI practice over RPT in gait speed can be further explained throughout un-altered stride length and both single and double support periods that significantly deteriorated only in the CON group. The latter results support the previous findings, which showed reduced stride length and cadence, less total knee motion, and increased double support time, especially for the operated side, when compared to uninvolved limbs in healthy control groups.[61,62]. Hence, our results suggest that adding MI training to RPT may be a suitable tool in continuing post-operative rehabilitation in a home environment in order to preserve functional ability following hospital discharge.

At the end, some limitations to the present study must be outlined. At first, a considerable number of initially randomized patients were unable to participate in final measurements due to reasons that were not related to the study. The great heterogeneity of orthopaedic patients is common and has recently been reported in another study.[17] Secondly, a relatively short follow-up period does not allow for any further conclusions regarding the long-term effectiveness of MI training on TKA patients’ physical capabilities. Future studies should, therefore, evaluate long-term lasting effects, which are crucial for successful rehabilitation and improving patients’ levels of independence in daily activities. The LEFS questionnaire used in the current study was successfully translated and validated for Slovenian-speaking community, however, the paper is still in the production phase which can be seen as limitation. Finally, due to rigorous inclusion criteria, the results cannot be generalized to the greater patient population (e.g., those individuals above 85 years of age, with more than 40 kg/m^2^ of BMI, with more comorbidity conditions, etc.).

## Conclusions

MI training, when added to RPT, led to improvements in multiple measures of patients’ physical functional capabilities following four weeks of self-administered practice in a home-based environment. Mainly, the results confirmed both the primary and secondary hypotheses: i) less deteriorated maximal isometric extension strength of the operated knee; ii) which has a positive influence on more functional tasks, such as chair sit-to-stand motion, as well as on gait speed ability under single- and dual-task conditions, respectively. However, MI was not superior to CON in improving patients’ self-reported physical capabilities. To conclude, MI training seems to be an applicable adjunct therapeutic tool to RPT for improving the objectively measured physical capabilities of TKA patients in the early post-operative period.

## Supporting information

S1 File CONSORT Checklist. (PDF)

S2 File Study protocol in original (Slovenian) language. (PDF)

S3 File Study protocol in English language. (PDF)

S4 File List of all outcome measures assessed in PhD project. (PDF)
