# Supplementary material for: Specific and general adaptations following motor imagery practice focused on muscle strength in total knee arthroplasty rehabilitation: A randomized controlled trial
Source: PLoS One. 2019 Aug 14;14(8):e0221089. doi: 10.1371/journal.pone.0221089 (PMC6693761; doi:10.1371/journal.pone.0221089)
Supplement: S2 File — (PDF) [file pone.0221089.s002.pdf]

Delovni naslov: ***Vpliv motorične predstave na izbrane funkcionalne parametre pacientov po popolni zamenjave (endoproteza) kolena***

Opis raziskave

Raziskava je predvidena v Ortopedski Bolnišnici Valdoltra (OBV) v letu 2017.

Cilj raziskave je ugotoviti vpliv kognitivnega treninga oz. motorične predstave na psiho-fizični status pacientov po operacijskem posegu popolne zamenjave kolena (TKA).

Subjekti:

V raziskavo bo vključenih 50 pacientov ki jih bomo razdelili v dve uravnotežene skupine

- Eksperimentalna (25 pacientov)
- Kontrolna (25 pacientov)

Vključitveni kriteriji:

- Unilateralna TKA, sekundarno zaradi osteoartritisa
- Moški spol
- Starost med 50 in 75 let
- Dovoljenje za fizioterapevtsko obravnavo - rehabilitacijo po operaciji

Izključitveni kriteriji:

- Pacienti, ki so že imeli posege kot je TKA na isti nogi ki bo operirana
- Bilateralna TKA
- Pacienti s nevrološkimi problemi vključujoč infarkt, multiplo sklerozo ali Parkinsonovo bolezen.
- Pacienti z revmatoidnim artritisom ali aktivnim rakavim obolenjem.
- Pacienti, ki so predhodno imeli izkušnje z treningom motorične predstave (MI).
- Krvavitev po operaciji, tromboze, pljučne bolezni.
- Splošna anestezija.

Potek študije:

Pacienti (n=50) bodo randomizirani v dveh skupinah: eksperimentalno (MIex) in kontrolno (CON) - 25 subjektov v vsaki skupini.
Vsi subjekti, ne glede na skupino, bodo imeli konvencionalno fizioterapijo po TKA, s tem da bo MIex imela ob klasični rehabilitaciji dodatno vadbo motorične predstave. Pacienti bodo aktivno sodelovali v fizikalni terapiji pod nadzorom licenciranega fizioterapevta OBV (vsebina in pogostost fizioterapevtske vadbe je že določena skladno s standardno obravnavo TKA, ki bo upoštevana). MIex bo imela vsak dan dva MI treninga – A- neposredno pred konvencionalno fizikalno terapijo, B- po večerji pred počitkom. Vsak MI trening bo trajal cca 20 minut in bo razdeljen v dva dela:

- *Prvi del bo zajemal 2 min gledanja posnetkov hoje na različnih podlagah; z in brez pripomočkov (zaradi doseganja motorične predstave giba); sledi 5 min predstavljanje iste naloge – gibalne vadbe v mislih, brez vidne prisotne mišične aktivacije.*

- *V drugem delu bodo pacienti 2 min gledali posnetke izvajanja izometrične mišične kontrakcije na dinamometru kjer se bodo izvajale meritve maksimalne želene izometrične mišične kontrakcije (MViC); sledi predstava izvedbe istega giba.*
  Subjekti si bodo predstavljali 50 MViC, razdeljeno v dve serije po 25 ponovitev. Intenzivnost kontrakcije bo 100% MViC z zadržkom 5 sekund. Med vsako kontrakcijo bo 5 sekund počitka in med vsako peto kontrakcijo 30 sekund počitka. Med serijami bo do maksimalno dve minuti odmora.

Po odpustu iz bolnišnice bodo pacienti izročeni video posnetki in opisi kognitivnih vaj s katerimi bodo nadaljevali v domači oskrbi. Ob tem bodo vodili dnevnik dela o dnevnem izvajanju vaj (manj kot 90% izvedba predvidenih vaj bo izključitveni kriteriji iz študije).

Meritve:

Pred TKA posegom, pred odhodom pacientov v toplice (4-5 tednov po posegu) in tri mesece po posegu bomo izvedli antropometrične meritve, raven bolečine (Visual analogue scale – VAS) in funkcionalni status z pomočjo naslednjih testov:

- Amplituda giba s pomočjo goniometra
- Maksimalna izometrična moč
- Aktivacijski vzorec skeletnih mišic (elektromiografija/EMG)
- Kontraktilne značilnosti in sestavo skeletnih mišic (tenziomiografija/TMG)
- Test agilnosti in dinamičnega ravnotežja (vstani in pojdi test/timed up to go-TUG)
- Vstani sedi (bilateralna pritiskovna plošča)
- Test hoje (Opto gait)
- Poraba kisika med hojo (COSMED K4b2)
- Vprašalnik (Oxford Knee Score/OKS)

Tabelarni prikaz poteka študije

**Bloki**	*Začetne meritve (prej TKA)*	*Prilagajanje nas tretma*	*Po prvemu bloku vadbe (4-5 tednov po TKA posegu)*	*3 mesece po TKA posegu*
**Meritve**	Testi		Testi	Testi
**Tretma**		MI	Fizikalna terapija	
**Vključene skupine**	MIex and CON	MIex	MIex and CON	MIex and CON

Predlagatelj raziskave:

Prof. dr. Rado Pišot

Znanstveno-raziskovalno središče Koper
