# Supplementary material for: Specific and general adaptations following motor imagery practice focused on muscle strength in total knee arthroplasty rehabilitation: A randomized controlled trial
Source: PLoS One. 2019 Aug 14;14(8):e0221089. doi: 10.1371/journal.pone.0221089 (PMC6693761; doi:10.1371/journal.pone.0221089)
Supplement: S3 File — (PDF) [file pone.0221089.s003.pdf]

Working title: Effects of motor imagery practice on selected functional parameters of patients after total knee arthroplasty

Study description

The study is planned to be conducted in Ordopedic Hospital Valdoltra (OBV) in 2017.

The aim of the research is to determine the impact of cognitive training i.e., motor imagery practice on the psycho-physical status of patients after a total knee arthroplasty (TKA).

Subjects:

In total 50 patients will be recruited in the study. They will be randomly assigned into:

- Experimental group (25 patients)
- Control group (25 patients)

Inclusion criteria:

- Unilateral TKA, secondary to osteoarthritis.
- Males.
- Aged between 50 and 75 years.
- Those who have permission for physiotherapeutic therapy i.e., post-operative rehabilitation.

Exclusion criteria:

- Patients, who are scheduled for revision TKA on the same side.
- Bilateral TKA.
- Patients with neurologic issues including myocardial infarct, multiple sclerosis or Parkinson's Disease.
- Patients with rheumatoid arthritis or active cancer.
- Patients, who have previous experience with motor imagery practice.
- Bleeding after surgery, thrombose, pulmonary diseases.
- General anesthesia.

Study protocol:

Patients (n = 50) will be randomized into two groups: experimental (MIex) and control (CON) – 25 subjects in each group.

All subjects, regardless of assigned group, will have conventional physiotherapy following TKA, with note that MIex will have additional motor imagery practice. Patients will actively participate in physical therapy sessions under the supervision of the licensed OBV physiotherapist (the content and frequency of physiotherapeutic exercise is already determined in accordance with the standardized TKA treatment that will be taken into account). MIex will have two MI training days - A- immediately before conventional physical therapy, B- after a dinner before resting. Each MI training will last approximately 20 minutes and will be divided into two parts:

- The first part will consider 2 minutes of watching videos of walking patterns on different surfaces; with and without gadgets (in order to achieve a better motor representation of the movement); followed by 5 minutes of imagining the same task - exercise in the mind, without overt muscle action.

- In the second part, patients will watch videos of performing isometric muscle contraction on a dynamometer for 2 minutes i.e., the same one where the measurements of the maximum voluntary isometric contraction (MViC) will be performed; followed by the imagining of the same movement.

Patients will imagine 50 MViC, divided into two series after 25 repetitions. The intensity of the contraction will be 100% MViC that should be held for 5 seconds. Following every MViC, there will be 5 seconds of rest, and following each fifth contraction, 30 seconds of rest. Inter set interval will be set on maximum of two minutes.

After being discharged from the hospital, patients will be supplied with videos and descriptions of cognitive exercises that they should perform at their homes. At the same time, they will keep a logbook on the daily exercise of exercises (less than 90% of the implementation of the planned exercises will be exclusionary criteria from the study).

Measurements:

Before the TKA interventions, one month after the intervention (4-5 weeks after TKA) and three months after TKA, we will perform anthropometric measurements, Visual analogue scale (VAS) and functional status using the following tests:

- *Flexibility of knee joint* (flexion and extension ROM) – assessed by manual goniometer
- *Maximal voluntary isometric strength* (MViC) (isometric dynamometer S2P)
- Activation patterns of skeletal muscles (electromyography/EMG)
- *contractile muscle parameters* (Tensiomyography)
- *electromechanical efficiency index* (Tensiomyography and M-wave module)
- *agility and dynamic balance* (Timed “Up & Go” Test)
- *Sit to stand (bilateral force plate)*
- Walking ability test (OPTOGait)
- Oxygen consumption test (COSMED K4b2)
- Questionnaire (Oxford Knee score/OKS)

Blocks	***PRE Baseline measurements (before TKA)***	***Addaptations to the treatmant***	***POST One month post TKA***	***POST Three months post TKA***
**Measurement assessments**	Tests		Tests	Tests
**Treatment**		MI	Physical therapy	
**Groups included**	MIex and CON	MIex	MIex and CON	MIex and CON

Research proposed by:

Prof. Dr. Rado Pišot

Science and Research Centre Koper
