# Supplementary material for: Specific and general adaptations following motor imagery practice focused on muscle strength in total knee arthroplasty rehabilitation: A randomized controlled trial
Source: PLoS One. 2019 Aug 14;14(8):e0221089. doi: 10.1371/journal.pone.0221089 (PMC6693761; doi:10.1371/journal.pone.0221089)
Supplement: S4 File — (PDF) [file pone.0221089.s004.pdf]

***Measurement assessments*:**

I. Anthropometry:

- Body weight and height (Stable stadiometer Seca, Birmingham, UK) and body composition (Bio-impedance system MALTRON BF-906).

II. Functional status:

- *flexibility of knee joint* (flexion and extension ROM) – assessed by manual goniometer
- *agility and dynamic balance* (Timed "Up & Go" Test)
- *Sit to stand (30 seconds)*
- *spatio-temporal gait parameters (STGP)* during different gait velocities will be measured by OPTOGAIT system (Microgate, Bolzano, Italy) under single- and dual-task conditions.
- *Hand grip strength along with both the knee extensors and flexors muscles maximal voluntary isometric strength* (MViC) (isometric dynamometer S2P)
- *Self - reported functional assessment* (OKS and LEFS questionnaires)

III. Muscle function:

- *contractile muscle parameters* (Tensiomyography)
- *electromechanical efficiency index* (Tensiomyography and M-wave module)
- *Voluntary activation level (using double interpolated twitch technique)*

IV. Pain level - Visual analogue scale (VAS)
